# Crystal structure of μ-fluorido-bis­{(η^4^-cyclo­octa­diene)[hexa­fluorido­anti­monato(V)]platinum(II)} hexa­fluorido­anti­monate(V) hydrogen fluoride 0.75-solvate^1^

**DOI:** 10.1107/S2056989015022835

**Published:** 2016-01-01

**Authors:** Konrad Seppelt, Roland Friedemann

**Affiliations:** a Freie Universität Berlin, Institut für Chemie und Biochemie – Anorganische Chemie, Fabeckstrasse 34-36, D-14195 Berlin, Germany

**Keywords:** crystal structure, cyclo­octa­diene complex, binuclear platinum complex, anhydrous hydrogen fluoride, superacid

## Abstract

In the complex cation of the binuclear solvated title salt, [Pt_2_F(SbF_6_)_2_(C_8_H_12_)_2_]SbF_6_·0.75HF, an F atom bridges the two platinum(II) atoms with a bond angle of 123.3 (2)°. The corresponding Pt—F bond lengths are in the range of other fluorine-bridged binuclear platinum(II) complexes. Two of the three SbF_6_^−^ anions each coordinate with one F atom to one platinum(II) atom. Including the η^4^-bound cyclo­octa­diene (COD) ligands, the overall coordination sphere of each platinum(II) atom is square-planar. The third SbF_6_^−^ anion is not bound to the complex. Hydrogen fluoride is present in the crystal structure as a solvent disordered over three positions, each with an occupancy of 0.25. F⋯F distances of 2.5512 (7), 2.6076 (8) and 3.2215 (10) Å to surrounding SbF_6_^−^ anions are indicative of F—H⋯F hydrogen-bonding inter­actions although no H atoms could be localized for the disordered solvent mol­ecules. The resulting hydrogen-bonded network is three-dimensional.

## Abstract 2

In the cation of the title compound, [Pt_2_(COD)_2_F(SbF_6_)_2_]SbF_6_·0.75HF, a fluorine atom bridges two platinum atoms. Each platinum atom is furthermore surrounded by a COD ligand and one fluorine atom of the octa­hedral SbF_6_ anion.

## Chemical context   

Platinum complexes of cyclic dienes, like cyclo­octa­diene (COD), are widely used in metal-organic chemistry to introduce new ligands by substitution of the diene. For instance, [Pt(CH_3_)_2_(COD)] is a commercially available staring material for most of the dimethyl complexes of platinum(II). Methyl ligands in platinum complexes can be protonated in superacids and eliminated as methane qu­anti­tatively. With anhydrous hydrogen fluoride (aHF), one or both methyl groups are protonated and replaced by a fluoride ion, but the resulting products cannot be crystallized because the formed fluoride ion does not sufficiently stabilizes the platinum complexes. With larger counter-anions like BF_4_^−^, AsF_6_^−^ or SbF_6_^−^, stable crystalline complexes can be formed and isolated (Friedemann & Seppelt, 2013 ▸). 
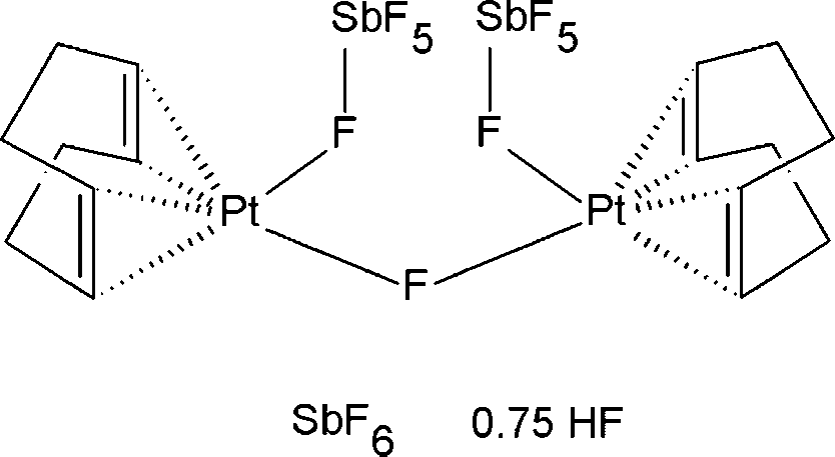


One methyl group of [Pt(CH_3_)_2_(COD)] reacts with aHF at low temperature under formation of methane; the second methyl group can be eliminated by the addition of anti­mony penta­fluoride. The resulting dissolved complex is stable at room temperature and can be crystallized by cooling to 200 K. The formed title compound [Pt_2_(COD)_2_F(SbF_6_)_2_]SbF_6_·0.75HF dissolves unreacted only in aHF or aceto­nitrile. With other organic solvents, a reaction takes place to form black undefined oils; with chlorinated solvents chlorido-platinum complexes are formed instead.

## Structural commentary   

**Figure 1:**
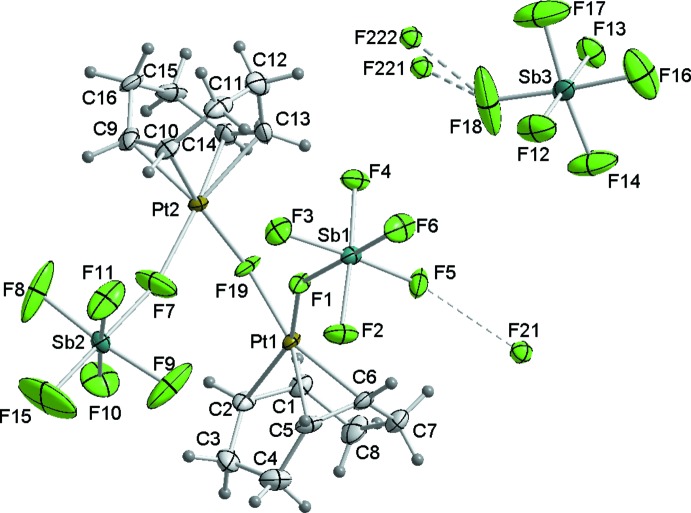
The structure of the mol­ecular entities of the title compound, with displacement ellipsoids drawn at the 50% probability level. Hydrogen bridges are marked with dashed lines.

Each of the two independent platinum(II) atoms is surrounded by one COD ligand in a double π-coordination, one fluorine atom of a SbF_6_^−^ anion and one bridging fluorine atom, resulting in a slightly distorted square-planar coordin­ation sphere (Fig. 1 ▸). The fluorine atom F19 bridges the two platinum(II) atoms with a bond angle of 123.3 (2)°. The corresponding Pt—F bond lengths [2.085 (4) Å and 2.065 (4) Å] are in the range of other fluorine-bridged binuclear platinum complexes [Pt—F 2.030 (9)–2.083 (10) Å; Friedemann & Seppelt, 2013 ▸) and somewhat longer than in non-bridging complexes like [PtF_2_(PPh_3_)_2_] [Pt—F = 1.999 (2) and 2.016 (2) Å; Yahav *et al.*, 2005 ▸). The two PtF_2_ planes are twisted by 69.8 (3)°. The third SbF_6_^−^ anion is not bonded to the complex. The COD ligands are bonded much stronger to the platinum(II) atoms than in the starting compound [Pt(CH_3_)_2_(COD)] (Smith *et al.*, 2000 ▸). This leads to shorter Pt—C bond lengths by up to 0.1 Å and an elongation of the olefinic bonds. The bite angles of the chelating ligands [88.85 (1)° at Pt1, 89.05 (1)° at Pt2] are close to the ideal 90° of a square-planar Pt^2+^ complex.

## Supra­molecular features   

**Figure 2:**
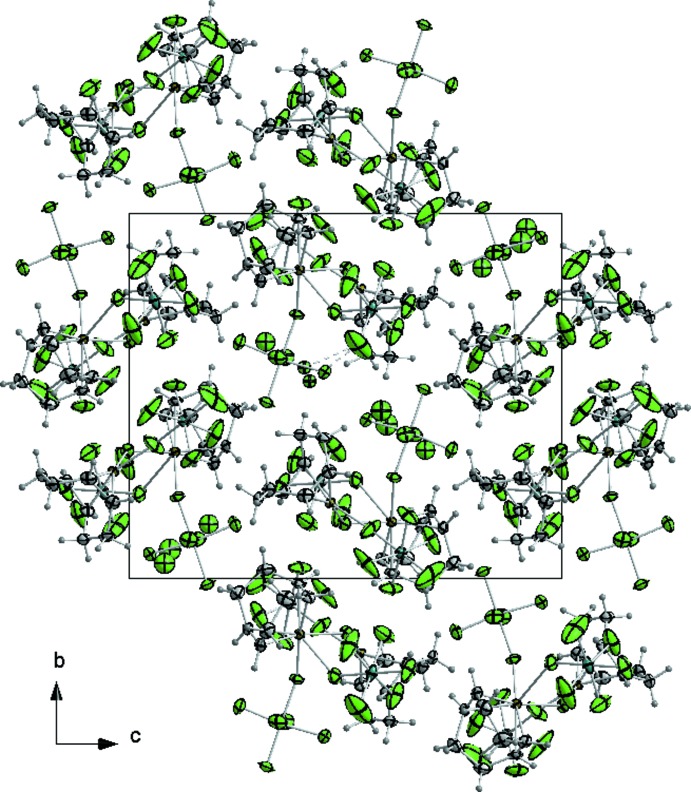
The crystal packing of the title compound in a view along [100].

The [Pt_2_(COD)_2_F(SbF_6_)_2_] cations and SbF_6_ anions are packed in such a way that voids are generated that are filled with disordered HF solvent mol­ecules (F21, F221 and F222). The shortest distances of these atoms to fluorine atoms of the surrounding SbF_6_^−^ anions [F221⋯F18 2.5512 (7), F222⋯F18 2.6076 (8) and F21⋯F5 3.2215 (10) Å] are in the typical range of F—H⋯F donor acceptor distances, marked in Fig. 1 ▸ with dashed lines. The packing of the mol­ecular entities in the crystal structure is shown in Fig. 2 ▸.

## Synthesis and crystallization   

[Pt(CH_3_)_2_(COD)] (40 mg, 0.12 mmol) and anti­mony(V) fluoride (80 mg, 0.36 mmol) were filled separated in a two chamber PFA tube. Anhydrous HF (0.5 ml) was condensed on it at 77 K. By heating to 200 K and mixing, a gas and a yellow solid were formed. The solid dissolved at room temperature under a second gas formation to a give clear yellow solution. The gas was removed and the sealed tube was slowly cooled to 200 K to form yellow single crystals of the title compound. NMR in aHF at room temperature: ^1^H *d*: 2.02 (*m*, *br*, 4H), 2.61 (*m*, *br*, 4H), 5.73 (*s*, 4H, ^2^ *J*_H,Pt_ = 95 Hz). NMR in CD_3_CN at room temperature: ^1^H *d*: 2.44 (*m*, *br*, 4H), 2.75 (*m*, *br*, 4H), 6.17 (*s*, 4H, ^2^ *J*_H,Pt_ = 67 Hz); ^19^F *d*: 122 (*m*, *br*); ^13^C{^1^H} *d*: 31.4 (*s*), 109.9 (^1^ *J*_C,Pt_ = 162 Hz); ^195^Pt{^19^F} *d*: −3424 (*s*).

## Refinement   

Crystal data, data collection and structure refinement details are summarized in Table 1 ▸. H atom positions of the COD ligand were refined with calculated positions in a riding model with C—H = 0.97 and 0.98 Å and *U*_iso_(H) = 1.2*U*_eq_(C). Atoms F21, F221 and F222 that are associated with the hydrogen fluoride solvent are disordered and were refined isotropically. Their occupation factors were fixed to 0.25 for each of these atoms which showed the best results in terms of reliability factors and *U*_iso_ values. Hydrogen atoms bound to the disordered solvent F atoms could not be detected and were consequently not considered in the final model. Some F atoms of the SbF_6_^−^ anions exhibited somewhat elongated ellipsoids. Since consideration of a split atom model had a negative effect (parts of these atoms could then only be refined isotropically), all F atoms of the SbF_6_^−^ anions were not refined as being disordered.

## Supplementary Material

Crystal structure: contains datablock(s) I. DOI: 10.1107/S2056989015022835/wm5233sup1.cif

Structure factors: contains datablock(s) I. DOI: 10.1107/S2056989015022835/wm5233Isup2.hkl

CCDC reference: 1439460

Additional supporting information:  crystallographic information; 3D view; checkCIF report

## Figures and Tables

**Table 1:** Experimental details

Crystal data	
Chemical formula	[Pt_2_F(SbF_6_)_2_(C_8_H_12_)_2_]SbF_6_·0.75HF
*M*_r_	1347.77
Crystal system, space group	Monoclinic, *P*2_1_/*c*
Temperature (K)	133
*a*, *b*, *c* (Å)	11.325 (4), 15.101 (6), 18.273 (7)
β (°)	100.61 (3)
*V* (Å^3^)	3071.7 (19)
*Z*	4
Radiation type	Mo *K*α
μ (mm^−1^)	11.81
Crystal size (mm)	0.10 × 0.10 × 0.02

Data collection	
Diffractometer	Bruker SMART CCD
Absorption correction	Multi-scan (*SADABS*; Bruker, 2006 ▸)
*T*_min_, *T*_max_	0.721, 1.000
No. of measured, independent and observed [*I* > 2σ(*I*)] reflections	47396, 9265, 7778
*R*_int_	0.041
(sin θ/λ)_max_ (Å^−1^)	0.716

Refinement	
*R*[*F* ^2^ > 2σ(*F* ^2^)], *wR*(*F* ^2^), *S*	0.038, 0.074, 1.10
No. of reflections	9265
No. of parameters	373
H-atom treatment	H-atom parameters constrained
	*w* = 1/[σ^2^(*F*_o_^2^) + (0.0136*P*)^2^ + 38.0407*P*] where *P* = (*F*_o_^2^ + 2*F*_c_^2^)/3
Δρ_max_, Δρ_min_ (e Å^−3^)	1.96, −1.66

Computer programs: *SMART* and *SAINT* (Bruker, 2006 ▸), *SHELXS97* (Sheldrick, 2008 ▸), *SHELXL2014* (Sheldrick, 2015 ▸), *DIAMOND* (Brandenburg, 2006 ▸) and *publCIF* (Westrip, 2010 ▸).

## supplementary crystallographic information

### Crystal data

**Table:**

[Pt_2_F(SbF_6_)_2_(C_8_H_12_)_2_]SbF_6_·0.75HF	*F*(000) = 2410
*M**_r_* = 1347.77	*D*_x_ = 2.912 Mg m^−^^3^
Monoclinic, *P*2_1_/*c*	Mo *K*α radiation, λ = 0.71073 Å
*a* = 11.325 (4) Å	Cell parameters from 999 reflections
*b* = 15.101 (6) Å	θ = 2.0–18.3°
*c* = 18.273 (7) Å	µ = 11.81 mm^−^^1^
β = 100.61 (3)°	*T* = 133 K
*V* = 3071.7 (19) Å^3^	Platelet, yellow
*Z* = 4	0.10 × 0.10 × 0.02 mm

### Data collection

**Table:**

Bruker SMART CCD diffractometer	7778 reflections with *I* > 2σ(*I*)
Radiation source: fine-focus sealed tube	*R*_int_ = 0.041
ω–scans	θ_max_ = 30.6°, θ_min_ = 1.8°
Absorption correction: multi-scan (*SADABS*; Bruker, 2006)	*h* = −14→16
*T*_min_ = 0.721, *T*_max_ = 1.000	*k* = −21→20
47396 measured reflections	*l* = −25→25
9265 independent reflections	

### Refinement

**Table:**

Refinement on *F*^2^	0 restraints
Least-squares matrix: full	Hydrogen site location: inferred from neighbouring sites
*R*[*F*^2^ > 2σ(*F*^2^)] = 0.038	H-atom parameters constrained
*wR*(*F*^2^) = 0.074	*w* = 1/[σ^2^(*F*_o_^2^) + (0.0136*P*)^2^ + 38.0407*P*] where *P* = (*F*_o_^2^ + 2*F*_c_^2^)/3
*S* = 1.10	(Δ/σ)_max_ = 0.001
9265 reflections	Δρ_max_ = 1.96 e Å^−^^3^
373 parameters	Δρ_min_ = −1.66 e Å^−^^3^

### Special details

**Table:**

Geometry. All e.s.d.'s (except the e.s.d. in the dihedral angle between two l.s. planes) are estimated using the full covariance matrix. The cell e.s.d.'s are taken into account individually in the estimation of e.s.d.'s in distances, angles and torsion angles; correlations between e.s.d.'s in cell parameters are only used when they are defined by crystal symmetry. An approximate (isotropic) treatment of cell e.s.d.'s is used for estimating e.s.d.'s involving l.s. planes.

### Fractional atomic coordinates and isotropic or equivalent isotropic displacement parameters (Å^2^)

**Table:**

	*x*	*y*	*z*	*U*_iso_*/*U*_eq_	Occ. (<1)
C1	0.9589 (6)	0.0517 (5)	0.5859 (4)	0.0249 (14)	
H1	0.9628	0.0430	0.5333	0.030*	
C2	0.8570 (6)	0.0145 (4)	0.6075 (4)	0.0242 (14)	
H2	0.8028	−0.0148	0.5666	0.029*	
C3	0.8479 (7)	−0.0224 (5)	0.6843 (5)	0.0349 (17)	
H3A	0.8909	−0.0782	0.6915	0.042*	
H3B	0.7643	−0.0342	0.6858	0.042*	
C4	0.8991 (8)	0.0407 (5)	0.7487 (4)	0.0386 (19)	
H4A	0.8557	0.0321	0.7892	0.046*	
H4B	0.9827	0.0260	0.7669	0.046*	
C5	0.8901 (6)	0.1385 (5)	0.7250 (3)	0.0256 (15)	
H5	0.8446	0.1757	0.7539	0.031*	
C6	0.9824 (6)	0.1840 (5)	0.6964 (4)	0.0254 (14)	
H6	0.9886	0.2470	0.7095	0.031*	
C7	1.0992 (6)	0.1443 (5)	0.6800 (5)	0.0344 (17)	
H7A	1.1544	0.1351	0.7266	0.041*	
H7B	1.1360	0.1864	0.6509	0.041*	
C8	1.0813 (7)	0.0558 (6)	0.6374 (5)	0.0384 (19)	
H8A	1.1441	0.0488	0.6082	0.046*	
H8B	1.0881	0.0074	0.6728	0.046*	
C9	0.3954 (6)	0.2266 (5)	0.4075 (4)	0.0285 (15)	
H9	0.3382	0.1819	0.4184	0.034*	
C10	0.4240 (6)	0.2919 (5)	0.4627 (4)	0.0257 (14)	
H10	0.3834	0.2847	0.5053	0.031*	
C11	0.4606 (7)	0.3856 (5)	0.4498 (4)	0.0347 (18)	
H11A	0.4960	0.4118	0.4972	0.042*	
H11B	0.3893	0.4195	0.4295	0.042*	
C12	0.5510 (8)	0.3930 (5)	0.3962 (4)	0.0357 (18)	
H12A	0.5075	0.3963	0.3454	0.043*	
H12B	0.5972	0.4471	0.4068	0.043*	
C13	0.6370 (7)	0.3136 (5)	0.4040 (4)	0.0287 (15)	
H13	0.7213	0.3297	0.4215	0.034*	
C14	0.6216 (7)	0.2364 (5)	0.3589 (4)	0.0264 (14)	
H14	0.6964	0.2095	0.3501	0.032*	
C15	0.5120 (8)	0.2173 (5)	0.2990 (4)	0.0342 (18)	
H15A	0.5108	0.1548	0.2866	0.041*	
H15B	0.5187	0.2504	0.2544	0.041*	
C16	0.3924 (7)	0.2421 (5)	0.3237 (4)	0.0322 (17)	
H16A	0.3749	0.3039	0.3124	0.039*	
H16B	0.3280	0.2071	0.2953	0.039*	
F1	0.7470 (3)	0.2768 (2)	0.6121 (2)	0.0244 (8)	
F2	0.7685 (5)	0.3520 (3)	0.7446 (2)	0.0412 (12)	
F3	0.5858 (4)	0.3941 (3)	0.6383 (3)	0.0354 (10)	
F4	0.7378 (4)	0.4343 (3)	0.5482 (2)	0.0342 (10)	
F5	0.9210 (4)	0.3940 (3)	0.6563 (3)	0.0364 (10)	
F6	0.7594 (5)	0.5180 (3)	0.6819 (3)	0.0435 (12)	
F7	0.5383 (6)	0.1340 (4)	0.5579 (4)	0.0685 (18)	
F8	0.3397 (5)	0.0409 (6)	0.5486 (4)	0.095 (3)	
F9	0.5896 (5)	0.1039 (5)	0.6986 (3)	0.081 (2)	
F10	0.5496 (6)	−0.0282 (3)	0.6053 (4)	0.0605 (16)	
F11	0.3781 (5)	0.1715 (4)	0.6448 (3)	0.0579 (17)	
F12	0.1200 (5)	0.2454 (4)	0.8549 (3)	0.0576 (16)	
F13	−0.0337 (5)	0.2311 (3)	1.0246 (3)	0.0420 (12)	
F14	−0.0805 (6)	0.1760 (5)	0.8855 (3)	0.073 (2)	
F15	0.3870 (9)	0.0075 (6)	0.6950 (6)	0.129 (4)	
F16	−0.0411 (7)	0.3453 (4)	0.9112 (4)	0.080 (2)	
F17	0.1630 (7)	0.3148 (7)	0.9912 (4)	0.106 (3)	
F18	0.1349 (9)	0.1414 (6)	0.9699 (5)	0.125 (4)	
F19	0.7447 (4)	0.1507 (3)	0.4971 (2)	0.0297 (9)	
Pt1	0.84029 (2)	0.15440 (2)	0.60610 (2)	0.01624 (5)	
Pt2	0.57666 (2)	0.20657 (2)	0.46502 (2)	0.01641 (5)	
Sb1	0.75340 (4)	0.40041 (3)	0.64879 (2)	0.01973 (9)	
Sb2	0.46054 (4)	0.06940 (3)	0.62808 (2)	0.02047 (9)	
Sb3	0.04406 (4)	0.23885 (4)	0.93973 (3)	0.03022 (11)	
F21	0.205 (2)	0.1481 (16)	0.1844 (13)	0.057 (6)*	0.25
F221	0.194 (3)	0.0405 (19)	0.0828 (15)	0.066 (7)*	0.25
F222	0.253 (3)	0.078 (2)	0.0949 (16)	0.075 (8)*	0.25

### Atomic displacement parameters (Å^2^)

**Table:**

	*U*^11^	*U*^22^	*U*^33^	*U*^12^	*U*^13^	*U*^23^
C1	0.021 (3)	0.027 (3)	0.027 (3)	0.007 (3)	0.004 (3)	−0.003 (3)
C2	0.025 (3)	0.013 (3)	0.032 (3)	0.006 (2)	−0.003 (3)	−0.002 (2)
C3	0.033 (4)	0.021 (4)	0.047 (5)	−0.002 (3)	0.001 (3)	0.005 (3)
C4	0.048 (5)	0.036 (4)	0.030 (4)	0.009 (4)	0.001 (3)	0.008 (3)
C5	0.032 (4)	0.025 (3)	0.017 (3)	0.008 (3)	−0.005 (3)	−0.001 (2)
C6	0.022 (3)	0.026 (3)	0.022 (3)	0.001 (3)	−0.010 (3)	−0.002 (2)
C7	0.021 (3)	0.038 (4)	0.040 (4)	0.000 (3)	−0.006 (3)	0.006 (3)
C8	0.022 (4)	0.036 (4)	0.053 (5)	0.012 (3)	−0.002 (3)	−0.003 (4)
C9	0.015 (3)	0.026 (4)	0.041 (4)	0.004 (3)	−0.003 (3)	0.002 (3)
C10	0.017 (3)	0.029 (4)	0.030 (3)	0.010 (3)	0.002 (3)	0.000 (3)
C11	0.039 (4)	0.018 (3)	0.040 (4)	0.010 (3)	−0.010 (3)	−0.005 (3)
C12	0.049 (5)	0.019 (3)	0.035 (4)	−0.004 (3)	−0.005 (3)	0.000 (3)
C13	0.027 (3)	0.030 (4)	0.030 (3)	−0.013 (3)	0.006 (3)	0.003 (3)
C14	0.027 (3)	0.030 (4)	0.023 (3)	−0.006 (3)	0.007 (3)	0.002 (3)
C15	0.053 (5)	0.029 (4)	0.017 (3)	0.005 (3)	−0.003 (3)	0.003 (3)
C16	0.029 (4)	0.031 (4)	0.029 (3)	0.003 (3)	−0.017 (3)	0.003 (3)
F1	0.0221 (19)	0.0154 (18)	0.032 (2)	0.0032 (14)	−0.0043 (16)	−0.0030 (15)
F2	0.055 (3)	0.044 (3)	0.023 (2)	0.008 (2)	0.001 (2)	0.0091 (19)
F3	0.022 (2)	0.039 (3)	0.046 (3)	0.0110 (19)	0.0093 (19)	0.000 (2)
F4	0.049 (3)	0.028 (2)	0.024 (2)	0.001 (2)	0.0008 (19)	0.0047 (17)
F5	0.020 (2)	0.033 (2)	0.053 (3)	−0.0063 (18)	−0.0014 (19)	−0.004 (2)
F6	0.060 (3)	0.022 (2)	0.043 (3)	0.006 (2)	−0.008 (2)	−0.0132 (19)
F7	0.086 (5)	0.063 (4)	0.066 (4)	0.008 (3)	0.038 (4)	0.037 (3)
F8	0.031 (3)	0.141 (7)	0.106 (5)	−0.006 (4)	−0.009 (3)	−0.093 (5)
F9	0.042 (3)	0.129 (6)	0.059 (4)	0.036 (4)	−0.025 (3)	−0.048 (4)
F10	0.071 (4)	0.028 (3)	0.085 (4)	0.019 (3)	0.021 (3)	−0.005 (3)
F11	0.041 (3)	0.056 (3)	0.070 (4)	0.027 (3)	−0.007 (3)	−0.034 (3)
F12	0.046 (3)	0.089 (4)	0.044 (3)	−0.019 (3)	0.025 (2)	−0.024 (3)
F13	0.056 (3)	0.043 (3)	0.031 (2)	0.011 (2)	0.018 (2)	0.004 (2)
F14	0.058 (4)	0.114 (6)	0.050 (3)	−0.052 (4)	0.017 (3)	−0.030 (4)
F15	0.144 (8)	0.127 (7)	0.147 (8)	0.026 (6)	0.108 (7)	0.085 (7)
F16	0.119 (6)	0.057 (4)	0.081 (5)	0.035 (4)	0.058 (4)	0.030 (3)
F17	0.077 (5)	0.167 (9)	0.073 (5)	−0.064 (5)	0.016 (4)	−0.057 (5)
F18	0.139 (8)	0.137 (8)	0.121 (7)	0.112 (7)	0.082 (6)	0.070 (6)
F19	0.024 (2)	0.030 (2)	0.027 (2)	0.0102 (17)	−0.0138 (16)	−0.0027 (17)
Pt1	0.01400 (10)	0.01497 (10)	0.01748 (10)	0.00341 (8)	−0.00306 (8)	−0.00149 (8)
Pt2	0.01658 (10)	0.01574 (10)	0.01592 (10)	0.00358 (9)	0.00038 (8)	0.00219 (8)
Sb1	0.0236 (2)	0.01486 (18)	0.01871 (18)	0.00293 (16)	−0.00145 (15)	−0.00242 (14)
Sb2	0.0211 (2)	0.0203 (2)	0.02157 (19)	−0.00094 (16)	0.00805 (16)	−0.00051 (15)
Sb3	0.0219 (2)	0.0445 (3)	0.0243 (2)	0.0015 (2)	0.00422 (17)	−0.0083 (2)

### Geometric parameters (Å, º)

**Table:**

C1—C2	1.404 (10)	C12—H12B	0.9700
C1—C8	1.527 (10)	C13—C14	1.419 (10)
C1—Pt1	2.128 (7)	C13—Pt2	2.146 (7)
C1—H1	0.9800	C13—H13	0.9800
C2—C3	1.530 (11)	C14—C15	1.523 (10)
C2—Pt1	2.121 (6)	C14—Pt2	2.141 (7)
C2—H2	0.9800	C14—H14	0.9800
C3—C4	1.541 (11)	C15—C16	1.551 (12)
C3—H3A	0.9700	C15—H15A	0.9700
C3—H3B	0.9700	C15—H15B	0.9700
C4—C5	1.537 (10)	C16—H16A	0.9700
C4—H4A	0.9700	C16—H16B	0.9700
C4—H4B	0.9700	F1—Sb1	1.980 (4)
C5—C6	1.428 (11)	F1—Pt1	2.142 (4)
C5—Pt1	2.155 (6)	F2—Sb1	1.876 (4)
C5—H5	0.9800	F3—Sb1	1.874 (4)
C6—C7	1.532 (11)	F4—Sb1	1.885 (4)
C6—Pt1	2.130 (6)	F5—Sb1	1.880 (4)
C6—H6	0.9800	F6—Sb1	1.873 (4)
C7—C8	1.540 (11)	F7—Sb2	1.946 (5)
C7—H7A	0.9700	F7—Pt2	2.132 (5)
C7—H7B	0.9700	F8—Sb2	1.854 (5)
C8—H8A	0.9700	F9—Sb2	1.837 (5)
C8—H8B	0.9700	F10—Sb2	1.876 (5)
C9—C10	1.406 (10)	F11—Sb2	1.856 (5)
C9—C16	1.543 (11)	F12—Sb3	1.909 (5)
C9—Pt2	2.148 (6)	F13—Sb3	1.923 (5)
C9—H9	0.9800	F14—Sb3	1.833 (5)
C10—C11	1.505 (10)	F15—Sb2	1.855 (6)
C10—Pt2	2.150 (6)	F16—Sb3	1.898 (6)
C10—H10	0.9800	F17—Sb3	1.883 (6)
C11—C12	1.546 (12)	F18—Sb3	1.823 (7)
C11—H11A	0.9700	F19—Pt2	2.065 (4)
C11—H11B	0.9700	F19—Pt1	2.085 (4)
C12—C13	1.534 (11)	F221—F222	0.87 (3)
C12—H12A	0.9700		
			
C2—C1—C8	122.9 (7)	C14—C15—H15B	109.1
C2—C1—Pt1	70.4 (4)	C16—C15—H15B	109.1
C8—C1—Pt1	113.2 (5)	H15A—C15—H15B	107.8
C2—C1—H1	114.2	C9—C16—C15	113.1 (5)
C8—C1—H1	114.2	C9—C16—H16A	109.0
Pt1—C1—H1	114.2	C15—C16—H16A	109.0
C1—C2—C3	126.9 (6)	C9—C16—H16B	109.0
C1—C2—Pt1	71.0 (4)	C15—C16—H16B	109.0
C3—C2—Pt1	110.6 (5)	H16A—C16—H16B	107.8
C1—C2—H2	113.4	Sb1—F1—Pt1	147.47 (19)
C3—C2—H2	113.4	Sb2—F7—Pt2	165.0 (4)
Pt1—C2—H2	113.4	Pt2—F19—Pt1	123.3 (2)
C2—C3—C4	113.3 (6)	F19—Pt1—C2	90.8 (2)
C2—C3—H3A	108.9	F19—Pt1—C1	92.8 (2)
C4—C3—H3A	108.9	C2—Pt1—C1	38.6 (3)
C2—C3—H3B	108.9	F19—Pt1—C6	158.6 (2)
C4—C3—H3B	108.9	C2—Pt1—C6	98.4 (3)
H3A—C3—H3B	107.7	C1—Pt1—C6	82.9 (3)
C5—C4—C3	112.5 (6)	F19—Pt1—F1	84.12 (15)
C5—C4—H4A	109.1	C2—Pt1—F1	154.5 (2)
C3—C4—H4A	109.1	C1—Pt1—F1	166.2 (2)
C5—C4—H4B	109.1	C6—Pt1—F1	95.1 (2)
C3—C4—H4B	109.1	F19—Pt1—C5	162.5 (2)
H4A—C4—H4B	107.8	C2—Pt1—C5	82.6 (3)
C6—C5—C4	123.3 (7)	C1—Pt1—C5	92.1 (3)
C6—C5—Pt1	69.6 (4)	C6—Pt1—C5	38.9 (3)
C4—C5—Pt1	112.5 (5)	F1—Pt1—C5	94.8 (2)
C6—C5—H5	114.4	F19—Pt2—F7	82.9 (2)
C4—C5—H5	114.4	F19—Pt2—C14	89.0 (2)
Pt1—C5—H5	114.4	F7—Pt2—C14	161.2 (3)
C5—C6—C7	126.9 (6)	F19—Pt2—C13	95.2 (2)
C5—C6—Pt1	71.5 (4)	F7—Pt2—C13	158.8 (3)
C7—C6—Pt1	108.8 (5)	C14—Pt2—C13	38.7 (3)
C5—C6—H6	113.7	F19—Pt2—C9	160.7 (2)
C7—C6—H6	113.7	F7—Pt2—C9	98.4 (3)
Pt1—C6—H6	113.7	C14—Pt2—C9	83.8 (3)
C6—C7—C8	113.6 (6)	C13—Pt2—C9	90.4 (3)
C6—C7—H7A	108.8	F19—Pt2—C10	161.0 (2)
C8—C7—H7A	108.8	F7—Pt2—C10	92.5 (3)
C6—C7—H7B	108.8	C14—Pt2—C10	100.4 (3)
C8—C7—H7B	108.8	C13—Pt2—C10	82.5 (3)
H7A—C7—H7B	107.7	C9—Pt2—C10	38.2 (3)
C1—C8—C7	111.6 (6)	F6—Sb1—F3	93.2 (2)
C1—C8—H8A	109.3	F6—Sb1—F2	94.4 (2)
C7—C8—H8A	109.3	F3—Sb1—F2	89.5 (2)
C1—C8—H8B	109.3	F6—Sb1—F5	92.8 (2)
C7—C8—H8B	109.3	F3—Sb1—F5	173.9 (2)
H8A—C8—H8B	108.0	F2—Sb1—F5	89.7 (2)
C10—C9—C16	124.4 (7)	F6—Sb1—F4	92.8 (2)
C10—C9—Pt2	71.0 (4)	F3—Sb1—F4	90.2 (2)
C16—C9—Pt2	110.5 (5)	F2—Sb1—F4	172.8 (2)
C10—C9—H9	114.3	F5—Sb1—F4	89.9 (2)
C16—C9—H9	114.3	F6—Sb1—F1	179.1 (2)
Pt2—C9—H9	114.3	F3—Sb1—F1	86.80 (18)
C9—C10—C11	125.5 (7)	F2—Sb1—F1	86.54 (19)
C9—C10—Pt2	70.8 (4)	F5—Sb1—F1	87.16 (18)
C11—C10—Pt2	108.9 (5)	F4—Sb1—F1	86.27 (18)
C9—C10—H10	114.3	F9—Sb2—F8	173.1 (4)
C11—C10—H10	114.3	F9—Sb2—F15	94.4 (5)
Pt2—C10—H10	114.3	F8—Sb2—F15	92.5 (5)
C10—C11—C12	113.6 (6)	F9—Sb2—F11	90.5 (3)
C10—C11—H11A	108.8	F8—Sb2—F11	90.0 (3)
C12—C11—H11A	108.8	F15—Sb2—F11	90.6 (4)
C10—C11—H11B	108.8	F9—Sb2—F10	89.3 (3)
C12—C11—H11B	108.8	F8—Sb2—F10	89.6 (3)
H11A—C11—H11B	107.7	F15—Sb2—F10	94.3 (4)
C13—C12—C11	111.6 (6)	F11—Sb2—F10	175.1 (3)
C13—C12—H12A	109.3	F9—Sb2—F7	85.5 (3)
C11—C12—H12A	109.3	F8—Sb2—F7	87.6 (4)
C13—C12—H12B	109.3	F15—Sb2—F7	179.7 (4)
C11—C12—H12B	109.3	F11—Sb2—F7	89.7 (3)
H12A—C12—H12B	108.0	F10—Sb2—F7	85.4 (3)
C14—C13—C12	125.4 (6)	F18—Sb3—F14	94.6 (5)
C14—C13—Pt2	70.5 (4)	F18—Sb3—F17	91.6 (5)
C12—C13—Pt2	112.3 (5)	F14—Sb3—F17	173.7 (4)
C14—C13—H13	113.6	F18—Sb3—F16	176.0 (5)
C12—C13—H13	113.6	F14—Sb3—F16	89.4 (4)
Pt2—C13—H13	113.6	F17—Sb3—F16	84.4 (4)
C13—C14—C15	124.7 (7)	F18—Sb3—F12	88.5 (3)
C13—C14—Pt2	70.8 (4)	F14—Sb3—F12	90.3 (2)
C15—C14—Pt2	108.3 (5)	F17—Sb3—F12	89.2 (3)
C13—C14—H14	114.7	F16—Sb3—F12	90.9 (3)
C15—C14—H14	114.7	F18—Sb3—F13	91.3 (3)
Pt2—C14—H14	114.7	F14—Sb3—F13	89.0 (2)
C14—C15—C16	112.6 (6)	F17—Sb3—F13	91.5 (3)
C14—C15—H15A	109.1	F16—Sb3—F13	89.3 (2)
C16—C15—H15A	109.1	F12—Sb3—F13	179.3 (2)
